# Supplementary material for: MetaRibo-Seq measures translation in microbiomes
Source: Nat Commun. 2020 Jun 29;11:3268. doi: 10.1038/s41467-020-17081-z (PMC7324362; doi:10.1038/s41467-020-17081-z)
Supplement: Supplementary file 10 — Supplementary Data 7 [file 41467_2020_17081_MOESM10_ESM.zip › File2/Confidence_VeryHigh_Taxonomy/84808_out.krona.html]

Javascript must be enabled to view this page.

members
magnitude
magnitudeUnassigned
count
unassigned
taxon
rank

84808\_out

7

2759
superkingdom
7

kingdom
7
4751

451864
7
subkingdom

5
phylum
5204

5302
5
subphylum

155619
class
5

452333
1
subclass

5338
1
order

5332
family
1

5333
1
genus

5334

SRS053356\_contig\_number\_contig-100\_1489.258776
species
1

order
4
452342

family
4
40420

4
genus
13562

13563
4
species

SRS013687\_contig\_number\_25522SRS023914\_contig\_number\_contig-100\_1727.162073SRS051031\_contig\_number\_34918SRS1041145\_contig\_number\_6364

phylum
2
4890

2
subphylum
147538

147550
class
1

subclass
1
222544

1
order
5139

35718
1
family

1
genus
5149

1
species
209285

1
varietas

SRS019685\_contig\_number\_contig-100\_1193.217201
285224

147541
1
class

1
subclass
451868

92860
order
1

715340
1
suborder

5020
1
family

55067

SRS076929\_contig\_number\_contig-100\_1790.149616
1
genus
